# Drug-Induced Enterocolitis Syndrome in Children: A Report of Two Cases

**DOI:** 10.7759/cureus.115039

**Published:** 2026-08-23

**Authors:** Helena Ferreira, Joana Capela, Ana Rita Araújo, Fernanda Teixeira, Diana Pinto

**Affiliations:** 1 Department of Pediatrics, Centro Materno Infantil do Norte, Unidade Local de Saúde de Santo António, Entidade Pública Empresarial, Porto, PRT; 2 Department of Pediatrics, Unidade Local de Saúde do Algarve, Faro, PRT; 3 Department of Pediatrics, Pediatric Allergology Unit, Centro Materno Infantil do Norte, Unidade Local de Saúde de Santo António, Entidade Pública Empresarial, Porto, PRT; 4 Department of Pediatrics, Pediatric Allergology Unit, Centro Materno Infantil do Norte, Centro Hospitalar Universitário do Porto, Porto, PRT

**Keywords:** amoxicillin, drug hypersensitivity reactions, drug-induced enterocolitis, drug provocation test, non–ige-mediated hypersensitivity

## Abstract

Drug-induced enterocolitis syndrome (DIES) is a rare non-IgE-mediated hypersensitivity reaction characterized by delayed-onset gastrointestinal symptoms, typically occurring one to four hours after drug ingestion. It shares clinical features with food protein-induced enterocolitis syndrome and remains underrecognized, with limited reported cases.

We present two pediatric cases of DIES triggered by amoxicillin. The first case involves an eight-year-old girl with allergic rhinoconjunctivitis and asthma, who experienced multiple episodes of vomiting and pallor following amoxicillin ingestion. A drug provocation test (DPT) with amoxicillin reproduced the reaction, with vomiting, lethargy, pallor, and diarrhea. The second case concerns a three-year-old girl, initially developing an urticarial rash after amoxicillin exposure at eight months of age. A subsequent DPT led to persistent vomiting, abdominal pain, and leukocytosis with neutrophilia, requiring intravenous fluid therapy. In both cases, DIES was diagnosed based on clinical criteria, and amoxicillin avoidance was recommended.

These cases align with previously reported pediatric DIES, reinforcing its association with amoxicillin. Laboratory findings, including leukocytosis, neutrophilia, and thrombocytosis, may support the diagnosis, but clinical recognition remains crucial, requiring high suspicion and increased awareness among clinicians. These cases highlight the diagnostic challenges in differentiating DIES from IgE-mediated drug hypersensitivity, the importance of conducting DPT in a controlled setting, and the need for further research on its pathophysiology, biomarkers, and prognosis.

## Introduction

Drug-induced enterocolitis syndrome (DIES) is a rare non-IgE-mediated hypersensitivity reaction characterized by gastrointestinal symptoms, typically occurring one to four hours after drug ingestion [1,2]. First described in 2014, DIES shares clinical features with food protein-induced enterocolitis syndrome (FPIES), a non-IgE-mediated food allergy with an analogous delayed presentation, including delayed-onset vomiting, diarrhea, pallor, lethargy, and, in severe cases, dehydration and hypovolemic shock [3,4]. The diagnosis is clinical and relies on major and minor criteria adapted from FPIES [4,5]. Fewer than 20 pediatric cases have been reported to date, most triggered by amoxicillin or amoxicillin-clavulanate, likely reflecting their widespread use in children [6,7]. Increasing awareness of this entity is essential to ensure timely recognition and appropriate management.

We present two pediatric cases of amoxicillin-induced DIES confirmed by supervised drug provocation test (DPT). Beyond adding to the limited case literature, both cases illustrate key knowledge gaps in DIES, the diagnostic overlap with IgE-mediated hypersensitivity, the uncertain patterns of cross-reactivity among beta-lactams, and the lack of long-term outcome data, as well as the importance of identifying safe alternative antibiotics.

## Case presentation

Case 1

An eight-year-old girl with allergic rhinoconjunctivitis, asthma, and a food allergy to hazelnuts was evaluated after her grandmother reported, during a routine consultation, episodes of vomiting and pallor occurring several hours after the child took amoxicillin or azithromycin, although she could not specify when these episodes had occurred. The symptoms were always self-limited and resolved without specific treatment after the antibiotic was withdrawn. Although the precise timing of these episodes could not be established retrospectively, their recurrent pattern after antibiotic exposure, in the absence of cutaneous or respiratory features, raised the suspicion of a non-IgE-mediated hypersensitivity reaction. Serum-specific IgE for both antibiotics was negative (<0.35 kU/L).

An open DPT with azithromycin was performed first and was uneventful. Three months later, an open DPT with amoxicillin was performed, using a full standard dose of 25 mg/kg without graded administration (total daily dose 50 mg/kg). Two and a half hours after administration, she developed persistent vomiting, initially of food content and later bilious, accompanied by marked lethargy, marked pallor, and three episodes of diarrhea, without cutaneous or respiratory symptoms. At baseline, vital signs were within normal limits (blood pressure 95/55 mmHg, heart rate 101 bpm, SpO₂ 98%, temperature 36.7°C, capillary refill time less than two seconds) and remained normal throughout the episode. Symptoms resolved after oral ondansetron, and no blood tests were obtained given the self-limited nature of the reaction. She was monitored for approximately seven hours until full clinical recovery, with no further symptoms thereafter. Based on the clinical presentation, a diagnosis of DIES was established.

Because cross-reactivity between beta-lactams could not be excluded, the patient was advised to avoid all beta-lactams until an alternative could be evaluated. As she had already tolerated azithromycin, this macrolide was retained as a safe option, and a subsequent open DPT with cefuroxime was uneventful, allowing the reintroduction of cephalosporins. Over approximately 18 months of follow-up, she received azithromycin on one occasion for an intercurrent infection, which was well tolerated.

Case 2

A three-year-old girl was referred to our Pediatric Allergy Unit for suspected amoxicillin hypersensitivity. At eight months of age, she had developed urticaria on the eighth day of amoxicillin treatment for pneumonia, which resolved with antihistamines and antibiotic withdrawal. No further exposure to aminopenicillins was documented between that episode and the referral to our unit. Serum-specific IgE for amoxicillin was negative (<0.35 kU/L).

An open-label DPT with amoxicillin was performed in our day-care unit using a full standard dose of 45 mg/kg, corresponding to a total daily dose of 90 mg/kg, without graded administration. Two hours and 50 minutes after administration, she developed diffuse abdominal pain, persistent vomiting, lethargy, and marked pallor, without any cutaneous or respiratory symptoms. On examination, the abdomen was soft and mildly tender on palpation, without guarding or rebound tenderness. Oral ondansetron was not tolerated and was therefore administered intravenously. Due to persistent vomiting, intravenous fluid therapy was initiated with a 10-mL/kg bolus of 0.9% sodium chloride followed by six hours of maintenance fluids, with full recovery. At baseline, vital signs were within normal limits (blood pressure 98/65 mmHg, heart rate 106 bpm, SpO₂ 98%, temperature 36.6 °C, capillary refill time less than two seconds) and remained normal throughout the episode. The patient was monitored for 10 hours until complete resolution, with no delayed symptoms. Blood tests performed during the acute reaction showed leukocytosis (26,220/µL; reference: 5,000-14,500/µL) with neutrophilia (23,230/µL; reference: 1,500-8,500/µL), thrombocytosis (628,000/µL; reference: 150,000-450,000/µL), a C-reactive protein level of 2.87 mg/L (reference: <5 mg/L), and a normal tryptase level (6.76 µg/L; baseline: 6.50 µg/L; reference: <11.4 µg/L). A diagnosis of DIES was established, and avoidance of amoxicillin was recommended. Since cefuroxime had been previously well tolerated, the patient was cleared to use cephalosporins. Over approximately 18 months of follow-up, she received cefuroxime on two occasions for intercurrent infections, both well tolerated.

## Discussion

Awareness of DIES remains low, and both its diagnostic tools and pathogenic mechanisms are still poorly understood [1]. Its true incidence is unknown: recent reviews, including an analysis of the FDA Adverse Event Reporting System, have identified only a limited number of well-characterized pediatric cases, most triggered by amoxicillin or amoxicillin/clavulanate [6,8]. This small number probably reflects underdiagnosis [9].

DIES shares similarities with FPIES, both involving a T-cell-mediated immune response, potentially triggered by drug metabolites interacting with the intestinal epithelium, leading to inflammation, disruption of the intestinal barrier, and increased neutrophilic activity [1,10].

The diagnosis of DIES remains clinical. Diagnostic criteria proposed by Van Thuijl et al., adapting those established for FPIES, require one major and at least three minor criteria [4,5]. Both of our patients developed vomiting within one to four hours of drug ingestion, without any cutaneous or respiratory symptoms, fulfilling the major diagnostic criterion. Additionally, both showed marked lethargy and pallor. In the first case, reexposure reproduced repetitive vomiting, and diarrhea also developed; in the second case, the provocation test represented the first objective enterocolitis episode, and intravenous fluid support was required. Both thereby fulfilled at least three minor criteria (Table 1).

**Table 1:** Application of the FPIES-derived diagnostic criteria for DIES to the two reported patients The diagnosis of DIES requires that a patient meets the major criterion and at least three minor criteria ^*^In Case 2, the initial reaction was urticaria; the DPT was thus the first objective enterocolitis episode DIES: drug-induced enterocolitis syndrome; FPIES: food protein-induced enterocolitis syndrome; DPT: drug provocation test Image credit: Adapted with permission from Van Thuijl et al. [4]

Criterion	Case 1	Case 2
Major criterion		
Vomiting 1-4 hours after drug ingestion, with the absence of classic IgE-mediated cutaneous or respiratory symptoms	Yes	Yes
Minor criteria (≥3 required)		
Second episode of repetitive vomiting on reexposure to the same drug	Yes	No^*^
Repetitive vomiting 1-4 hours after ingestion of a different drug	No	No
Extreme lethargy	Yes	Yes
Marked pallor	Yes	Yes
Need for emergency department visit	No	No
Need for intravenous fluid support	No	Yes
Diarrhea within 24 hours of ingestion	Yes	No
Hypotension	No	No
Hypothermia	No	No
Total minor criteria fulfilled	4	3

In the second case, cutaneous involvement was present only in the initial reaction. Its Day-8 latency and urticarial phenotype are atypical for DIES and more consistent with an infection-associated exanthem or a morbilliform drug eruption. In pediatric patients, skin manifestations arising during antibiotic treatment are frequently attributable to the underlying infection rather than to drug allergy.

The DIES phenotype became apparent only on DPT, with no skin involvement. This is consistent with reports that DIES may not manifest on first exposure and is typically not accompanied by cutaneous symptoms, although skin involvement has occasionally been described in initial reactions [1,3,11].

Differentiating DIES from nonallergic drug adverse effects or IgE-mediated hypersensitivity remains challenging. The absence of cutaneous or respiratory symptoms helps distinguish DIES from IgE-mediated drug hypersensitivity [2,12]. The key differences between DIES and drug-related adverse effects lie in severity, timing of onset, and the presence of associated systemic symptoms. Gastrointestinal drug adverse effects, although common, are typically mild, self-limiting, and dose-related, whereas DIES produces more severe gastrointestinal symptoms often requiring medical intervention [1].

Laboratory findings such as leukocytosis, neutrophilia, thrombocytosis, and normal tryptase levels, as observed in our second case, although not specific, may provide supportive evidence for DIES [1,6]. In a recent review, Arriba-Méndez et al. suggested including intense abdominal pain and neutrophilia as additional, minor diagnostic criteria, as these findings are frequently observed in DIES; both were present in our second case [7]. These proposed additions have not been validated and are not part of the criteria applied in Table 1. Both patients had negative specific IgE to amoxicillin, and the second patient showed no elevation in serum tryptase, findings consistent with previous reports [2,6].

Although not required for diagnosis, DPT is the most useful test to confirm or exclude DIES. Both provocations used a single full therapeutic dose, in accordance with our institutional protocol for suspected nonimmediate, low-risk hypersensitivity reactions without alarm features, based on recent international studies and guidelines supporting single-dose provocation in such cases [13]. Both were performed in a hospital setting equipped for continuous cardiorespiratory monitoring and immediate supportive care, with observation extending well beyond the expected one-to-four-hour latency. No specific protocol exists for suspected DIES; however, given the delayed onset and potential severity of these reactions, some authors advocate an approach similar to that used in FPIES, with fractionated doses and prolonged observation between steps, under close surveillance and with preestablished venous access, owing to the risk of hypotension and hypovolemia [7]. Although provocation entails deliberate reexposure, it remains the diagnostic gold standard for DIES; in our patients, it was undertaken only after informed consent from the legal guardians.

There is no standardized treatment protocol, and management is primarily supportive. As in FPIES, treatment relies on antiemetic therapy, particularly ondansetron and intravenous fluids [2,6]. Corticosteroids have not been shown to be effective but may be considered in severe cases [1].

In both of our patients, cefuroxime, a cephalosporin whose side chain differs from that of amoxicillin, was tolerated. Reported tolerance of penicillins (which lack the aminopenicillin side chain) and of cephalosporins with distinct side chains suggests that cross-reactivity in amoxicillin-induced DIES is driven by the aminopenicillin side chain rather than by the shared beta-lactam ring [1,7,11]. Avoidance of the entire class may therefore be unnecessary; however, evidence remains limited to a small number of reported cases, and tolerance should always be assessed individually [7].

This report has some limitations. The diagnostic criteria applied are adapted from FPIES and have not been formally validated for drug-induced reactions. In addition, part of the clinical history in the first case was retrospective and imprecise, with no electronic or pharmacy records available to corroborate it, so the diagnosis rested on the DPT rather than on the historical account alone. Finally, the follow-up period was relatively short, and long-term data on the natural history of DIES, including the possible development of tolerance to the culprit drug, are not available.

## Conclusions

These two cases add to the limited literature on DIES and aim to raise awareness of this condition. DIES should be considered in children presenting with delayed repetitive vomiting after antibiotic administration, in the absence of IgE-mediated skin or respiratory symptoms. Given the potential severity and delayed presentation of these reactions, DPT in suspected DIES warrants specific precautions, including a controlled hospital environment, prolonged monitoring, and venous access, in line with recent recommendations. Once the culprit drug is withdrawn, it is important to identify safe alternative antibiotics. Evidence on beta-lactam cross-reactivity in DIES is based on a limited number of cases, and tolerance to alternative beta-lactams should be assessed individually until larger studies are available. Future studies are needed to clarify the pathophysiology of DIES, identify reliable biomarkers, define patterns of cross-reactivity, and establish its long-term prognosis.
